# Estimating the Energy Costs of Intermittent Exercise

**DOI:** 10.2478/hukin-2013-0050

**Published:** 2013-10-08

**Authors:** Christopher B. Scott, Charles Fountaine

**Affiliations:** 1 Department of Exercise, Health and Sport Sciences; University of Southern Maine, USA.; 2 Department of Health, Physical Education and Recreation; University of Minnesota, Duluth, USA.

**Keywords:** resistance training, intense exercise, total energy expenditure, work volume

## Abstract

To date, steady state models represent the only acceptable methodology for the estimation of exercise energy costs. Conversely, comparisons made between continuous and intermittent exercise generally reveal major physiological discrepancies, leading to speculation as to why steady state energy expenditure models should be applied to intermittent exercise. Under intermittent conditions, skeletal muscle invokes varying aerobic and anaerobic metabolic responses, each with the potential to make significant contributions to overall energy costs. We hypothesize that if the aerobic-only energetic profile of steady state exercise can be used to estimate the energetics of non-steady state and intermittent exercise, then the converse also must be true. In fact, reasonable estimates of energy costs to work volumes or work rates can be demonstrated under steady state, non-steady state and intermittent conditions; the problem with the latter two is metabolic variability. Using resistance training as a model, estimates of both aerobic and anaerobic energy cost components, as opposed to one or the other, have reduced the overall energetic variability that appears inherent to brief, intense, intermittent exercise models.

## Introduction

Since the 1950s (Christensen, 1956) studies of intermittent exercise have taken place, with a seemingly limitless combination of exercise and recovery periods examined. For example, use of the term ‘intermittent exercise’ has ranged from forty repeated 15 m sprints with 30 s of recovery between bouts (Balsom et al., 1992), to 15 min periods of brisk walking with a between bout recovery period of 2 hours (Donnelly et al., 2000). Whether performed continuously or intermittingly, running and cycling are well known as aerobic-type exercise, thus treadmills and cycle ergometers populate most exercise science labs and are reflected heavily within the published research. Nonetheless, real world use of intermittent exercise is perhaps best demonstrated with resistance training, in which multiple sets of upper and lower body exercises with a compromised blood flow (oxygen delivery) are traditionally separated by defined rest and recovery periods (Edwards et al., 1972; Tamaki et al., 1994).

Through investigations of both single and multiple sets of resistance training, we have found the energetic profile of intermittent exercise to be the opposite of steady state models (Scott, 2006; Scott et al., 2009; Scott et al., 2011a; Scott et al., 2011b; Scott, 2012a; Scott, 2012b). Accordingly, our research has made four primary distinctions. First, the actual lifting time of a prescribed set of resistance training repetitions often takes seconds, resulting in minimal exercise oxygen uptake during the work bout; with steady state exercise, oxygen uptake represents the largest energetic component. Second, with resistance training, the anaerobic energetic component was always significant; with steady rate exercise, it does not need consideration. Third, after each resistance training set, oxygen uptake rises and peaks in recovery; steady state oxygen uptake peaks and plateaus during the exercise, falling exponentially in recovery. Fourth, when the recovery oxygen uptake periods between sets were separated from exercise oxygen uptake, they typically resulted in the largest component to overall energy costs; steady state exercise has a single less costly recovery component (excess post-exercise oxygen consumption, EPOC). Even with such discrepancies we hypothesize that the overall or total aerobic and anaerobic energy costs of intermittent exercise rise in proportion to a given work volume, just as it would for steady rate exercise. That is, if the aerobic-only energetic profile of steady state exercise can be used to estimate the energy costs of non-steady state and intermittent exercise, then the converse also must be true.

### Oxygen uptake and steady state exercise

**Figure 1:**
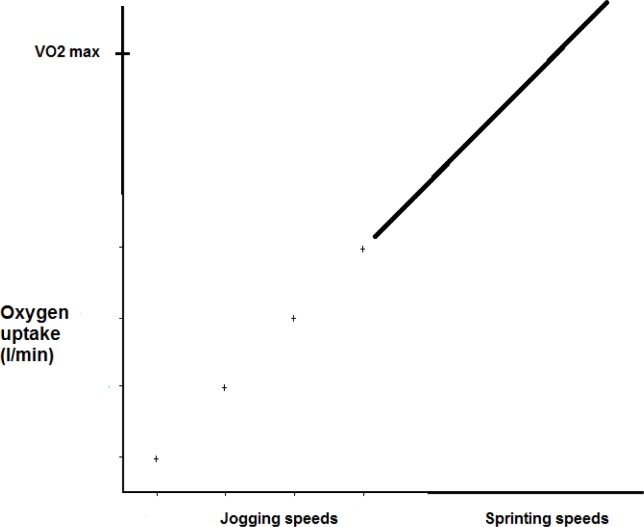
A linear extrapolation of low to moderate jogging energy expenditure at four specific speeds are used to estimate costs at sprinting speeds above the anaerobic threshold (and VO_2max_). Energy expenditure is represented exclusively as oxygen uptake.

We adopt the nomenclature of Reis et al. (2011) where steady state oxygen uptake defines ‘energy expenditure’, with the term ‘energy cost’ referring to a reasonable albeit variable estimate of overall energetics (aerobic and anaerobic). The gold standard relationship between energetics and exercise involves a steady rate power output matched to a steady state oxygen uptake. Based on this relationship, light to moderate exercise can be plotted along with the rate at which oxygen is consumed and then extrapolated linearly to greater work rates (Figure 1). In reality though, when reported as the expenditure of locomotion (energy expenditure per minute) or as expenditure of transport (energy expenditure per kilometer), linearity between steady state exercise and oxygen uptake is not found (Steudel-Numbers and Wall-Scheffler, 2009). This has not stopped, nor should it, the use of linear based equations that estimate the energetics of steady rate walking, jogging, or cycling (ACSM, 2014).

Oxygen uptake rates continue to rise when performing intense steady state exercise so that extra energy is involved, further contradicting linear models. Exercise scientists have denoted the terms ‘slow oxygen uptake component’ or ‘stage 3 oxygen uptake kinetics’ after identifying the phenomenon (Gaesser and Poole, 1996). Disparity or mismatch between steady rate power output and non-steady state oxygen uptake appears to accompany an altered recruitment pattern of skeletal muscle (Krustup et al., 2004) and/or a decrease in contractile efficiency (Zoladz et al., 2008). Exercise intensities above the anaerobic threshold clearly challenge the traditional steady state model. Both within and among subjects, movement economy as well as exercise intensity creates variability about a measurement of oxygen uptake for a given exercise.

### Oxygen uptake and non-steady state exercise

With ramping-type exercise stress tests, where work is continuously changing, Hughson and Inman (1986) revealed the inherent variability of oxygen uptake rates. Within-subject coefficient of variation (CV) ranged from 18.5 to 29.3%, making the case that cardiorespiratory responses cannot be properly assessed from a single ramp test to exhaustion (Swanson and Hughson, 1988). With such variability, the use of oxygen uptake to estimate the energy costs of non-steady state exercise must also be called into question.

**Figure 2:**
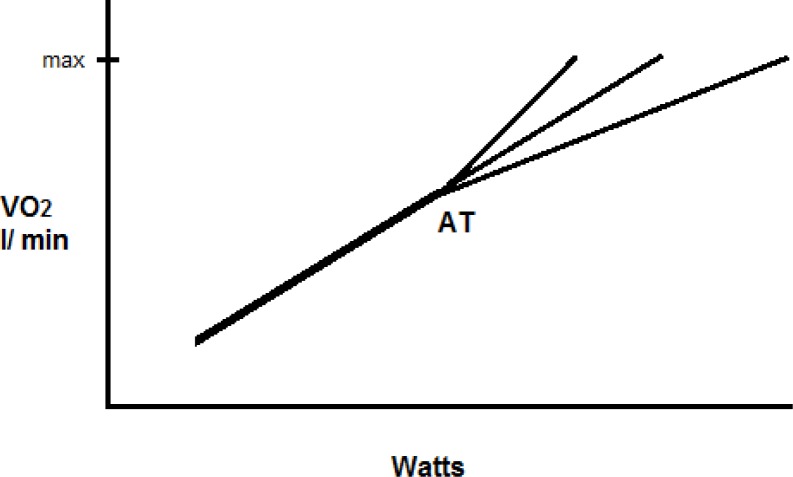
Results of the Hansen et al study (1988) are shown for a slow, moderate and fast ramping (cycling) test to exhaustion – the same patterns were seen both within and between subjects. Oxygen uptake rates are similar for all three tests below the anaerobic threshold (AT). After the AT is reached, the slow ramping protocol (∼16 min duration) has oxygen uptake rates that climb upward, the faster protocol (∼5 min duration) has suppressed oxygen uptake rates in comparison. With the moderate ramping protocol (∼9.5 min duration) an average of the slow and fast ramp tests is seen, with a linear response that mimics the steady state extrapolation of figure one (anaerobic energy costs were not estimated). With a compromised blood flow, weight lifting likely promotes a decrease in oxygen uptake rates by working skeletal muscle (mimicking the fast ramping protocol).

To the contrary, Wassermann and colleagues (1994) revealed a relatively stable increase in oxygen uptake with a cycling ramp test at 10.29 ml O2 min−1 Watt−1. Hansen et al. (1988) further examined how rate changes in power output affect oxygen uptake rates. When below the anaerobic threshold, no differences were found among slow, moderate or fast ramping tests to exhaustion. However, above the anaerobic threshold, the fastest ramping lowered oxygen uptake rates at a given power output while they increased with slower ramping (Figure 2). Moderate ramping continued to project a linear oxygen uptake rate to power output relationship, between the slow and fast protocols, both within and between subjects (i.e., the steady state model, Figure 1). Figure 2 can be viewed using the fast and slow oxygen uptake rates as “error bars” (i.e., variability) with the moderate oxygen uptake rate demonstrating a reasonable estimate of the energy cost central tendency for a variety of work rates above the anaerobic threshold.

Myers et al. (1989) used a ramping treadmill test to exhaustion and also found considerable variability in oxygen uptake rates over the course of the test among and within subjects tested on different days. The central tendency of this variability was 3.26 ml O2 per minute as work steadily increased. Twenty five years earlier, Margaria and colleagues (1963) noted a strikingly similar rise in oxygen uptake, for a series of steady rate treadmill tests at 3.3 ml O2 kg−1 min−1 per minute. It appears possible then, that an average oxygen uptake rate for a given exercise can be identified as a reasonable estimate of the aerobic energy cost component, though considerable variability is evident, indeed inherent, to intense non-steady rate exercise (Figure 2).

### Aerobic and anaerobic contributions to exercise

**Figure 3:**
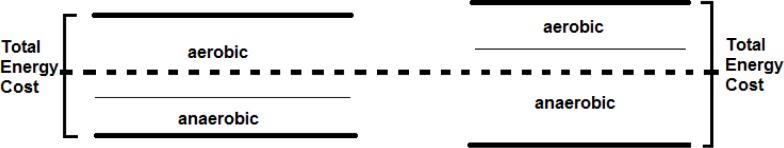
Anaerobic and anaerobic energy contributions are shown for two brief intense sets of identical exercise, both of which are required to represent overall energy costs. Note that for each set the extent of the aerobic and anaerobic metabolic components differs significantly (the high and low thick black lines can be viewed as “error bars”). Overall energy costs however are not significantly different as designated by the central tendency (dotted line).

Slower and faster oxygen uptake rates at a given work rate or work volume (within and among subjects) are likely to associate with the “recruitment” patterns of the aerobic and anaerobic metabolic systems. For example, for both trained and sedentary subjects, an estimation of total ATP turnover for a bout of two-leg knee extension exercise was similar, however the degree of the contributions of the aerobic and anaerobic metabolic systems between the two was different; a greater aerobic cost was associated with a decreased anaerobic cost, total energy costs were the same (Layec et al., 2009). In a comparison of treadmill sprinting and cycling with equated exercise, oxygen uptake and blood lactate differed for each yet total estimated costs were again the same (Scott et al., 2006). The concept is demonstrated with Figure 3: the central tendency of the overall or total cost is relatively constant, yet the contributions of the aerobic and anaerobic systems vary, often dramatically (both within and between subjects).

Adherence to steady state energetic models suggests *a priori* that blood lactate measurements cannot be used in the estimation of anaerobic energetics. In fact, historical studies reveal an energy cost rise with blood lactate measures *after* heavy to severe bouts of exercise that overlie the aerobic component *during* exercise in a predictable fashion, regardless of work rates (i.e., per minute) or work volume (e.g., over 5 to 30 second periods) (di Prampero and Ferretti, 1999; Margaria et al., 1963; Margaria et al., 1964). Margaria et al. (1963; 1964) cleverly matched both the aerobic and anaerobic component increases that were fitted to a single rate function scale with an energy cost of ∼3.0 ml of oxygen uptake per kilogram of body weight. Together with aerobic costs, the anaerobic costs of intermittent exercise increase in a rather specific manner, with each metabolic system complementing the other as part of an estimate of the overall or total energetics (figure 3). Like aerobic energy costs markers (i.e., oxygen uptake) for brief intense exercise, blood lactate can be a variable measure where the collection site (arteriole, capillary, venous) and time to peak – among other things – are influenced by the specificity of the exercise (Bishop and Martino, 1993).

### Intermittent exercise energy cost vector

**Figure 4:**
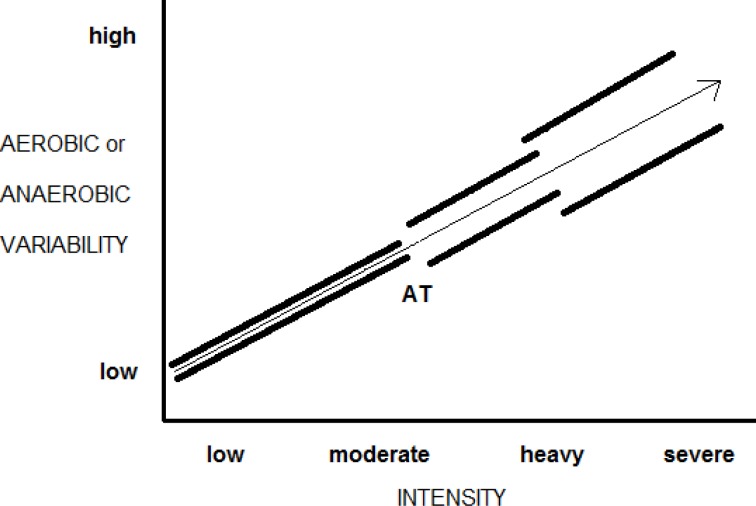
The extent of aerobic or anaerobic variability (figure 3) is shown as the distance between two thick black lines for any specific low to moderate, moderate to heavy and heavy to severe exercise rate. Variability also increases as exercise periods become shorter. If the X-axis indicated work volume and the Y-axis total energy costs, a vector (arrow) can be drawn throughout as the central tendency of the exercise cost.

Based on the aforementioned research a *vector* is proposed for a given type of intermittent exercise consisting of the direction/slope of the increasing energetic contribution with increases in work volume, along with the number of metabolic systems involved. The vector for any specific exercise should, at least hypothetically, overlie steady state oxygen uptake at low to moderate intensities for that particular exercise (Figure 1) and, the linear central tendency of those changes in aerobic and anaerobic energy cost contributions above the anaerobic threshold (Figure 4). Exercise to fatigue reveals a parallel complimentary energy cost relationship, a fixed cost added to but in proportion to non-fatiguing conditions (Scott and Earnest, 2011).

The steady state oxygen uptake rate of very low (steady rate) exercise has been extrapolated outwards in an attempt to foresee the aerobic and anaerobic energy costs of brief intense intermittent work. Robergs et al. (2007) did the former using the bench press exercise with loads between 5–23% of a one repetition maximum (1-RM). Five-minute lifting periods were measured and a steady state oxygen uptake plateau for each period was calculated and converted into a calorie per minute measurement. Scott et al. (2009) utilized an intermittent methodology to study the bench press (50% 1-RM), measuring separately exercise and recovery oxygen uptake (in liters as opposed to liters per minute). Peak blood lactate was used to estimate anaerobic energy contributions (di Prampero and Ferretti, 1999; Margaria et al., 1963; Margaria et al., 1964). Both studies had subjects lift and lower the weight at the same rate (1.5 seconds up, 1.5 seconds down). Whereas an indirect comparison, the estimated energy costs to work relationship for both studies was almost identical (Scott et al., 2009). If steady rate exercise models do in fact provide a reasonable estimate of intermittent energetics with heavy to severe work, then the reverse also appears true, albeit with one important caveat – aerobic and anaerobic metabolic variability (Figures 3, 4).

### Variability is the problem

A Smith weight lifting machine allows the lifter to move the bar only in a vertical plane. Under these conditions the major source of measurement variability is the vertical distance the weight lifting bar traveled as a subject lifts and lowers the weight. With eight subjects the coefficient of variation (CV) for triplicate measures of submaximal bench press work was about 5.0% (for 7, 14 and 21 repetitions) (Scott et al., 2009). However, the aerobic and anaerobic energetic CV’s were as high as 47%, and EPOC CV’s for all lifts was approximately 30%. Based on these data, the ‘economy’ of movement was relatively stable, but the inherent variability associated with each aerobic and anaerobic energy component was problematic. With such extensive variability, a conclusion could be made that *both* the aerobic and anaerobic energy estimates lack validity for brief intense intermittent exercise. For this same investigation, the aerobic and anaerobic CV dropped from a high of 47% each to as low as 15% when combined (Scott et al., 2009). The central tendency of an aerobic and anaerobic energy cost estimate helps provide a less variable assessment of overall or total energy costs for brief, intense intermittent exercise that hypothetically should follow steady state models (figure 4).

## Conclusion

We hypothesize that the overall or total aerobic and anaerobic energy costs of intermittent exercise rise in proportion to a given work volume, just as it would for steady rate exercise. Variability is an inherent part of estimating energy costs: at power outputs above the anaerobic threshold for steady rate exercise, for non-steady state exercise tests to exhaustion above the anaerobic threshold, and for bouts of intermittent exercise. The variability of energy cost estimates for resistance training has been reduced by 1) estimating aerobic *and* anaerobic energetic systems (exercise oxygen uptake, blood lactate, EPOC) and, 2) repeated measurements for any given subject and exercise using a measure of central tendency as the total energy cost estimate.
